# Pachydermodactyly: Soft Tissue Enlargement of the Fingers in a Teenager

**DOI:** 10.7759/cureus.41923

**Published:** 2023-07-15

**Authors:** Andrea Gallardo-Villamil, Luis E Cano-Aguilar, Estela Pérez-Muñoz, Mauricio Rojas-Maruri, Marimar Saez de Ocariz

**Affiliations:** 1 Dermatology, National Institute of Pediatrics, Mexico City, MEX; 2 Dermatology, General Hospital Dr. Manuel Gea González, Mexico City, MEX; 3 Pathology, National Institute of Pediatrics, Mexico City, MEX; 4 Dermatology and Pediatrics, National Institute of Pediatrics, Mexico City, MEX

**Keywords:** digital fibromatosis, fusiform swelling of the fingers, pediatric dermatology, soft tissue enlargement, pachydermodactyly

## Abstract

Pachydermodactyly (PDD) is an uncommon and benign digital fibromatosis of unknown etiology. It is characterized by a fusiform swelling of the medial and lateral sides of the fingers, with unspecific histopathological features of an increased number of fibroblasts, collagen, and mucin deposit in the dermis. Due to its rarity, PDD could be misdiagnosed as rheumatic arthropathies, which could lead to unnecessary immunosuppressant treatments. Here, we report the case of a 16-year-old boy who presented progressive and asymptomatic soft tissue enlargement of multiple fingers in both hands. The histopathological study and X-ray findings correlated with PDD diagnosis. Intralesional corticoid treatment was indicated with a mild improvement.

## Introduction

Pachydermodactyly (PDD) is a rare and unspecific digital fibromatosis [1] characterized by a symmetrical, chronic, and fusiform swelling of the fingers [2]. Its prevalence is still unknown, and there is still no standard diagnostic approach to evaluate affected patients. In 2015, Chen et al. [3] proposed six diagnostic criteria to improve its related diagnostic delay: 1) asymptomatic patient, 2) absence of morning stiffness, 3) absence of symptoms during palpation, 4) ulnar or radial finger swelling, 5) unremarkable test results, and 6) soft tissue swelling on plain radiographs. Nevertheless, Vázquez Fernández et al. [1] performed the first PPD systematic review and stated a mean diagnostic delay of three years. The typical patient was described as a young male with bilateral and asymptomatic ulnar swelling of the fingers, with a history of mechanical injury in 50% of patients, and no other family members affected. The authors suggested that skin biopsy is necessary in patients who do not meet the diagnostic criteria proposed by Chen et al. [3]. It is important to exclude any rheumatologic entities that could delay its indicated treatment, such as ending any mechanical trauma and intralesional corticoids in early stages in patients with aesthetic concerns due to their effect on the collagen synthesis [1]. There are only a few reported cases of surgical treatment for PPD that could be related to the morbidity associated if the surgery is performed in all the affected fingers. Here, we report a case of PDD that presented a mild improvement with intralesional corticosteroid treatment.

## Case presentation

**Figure 1:**
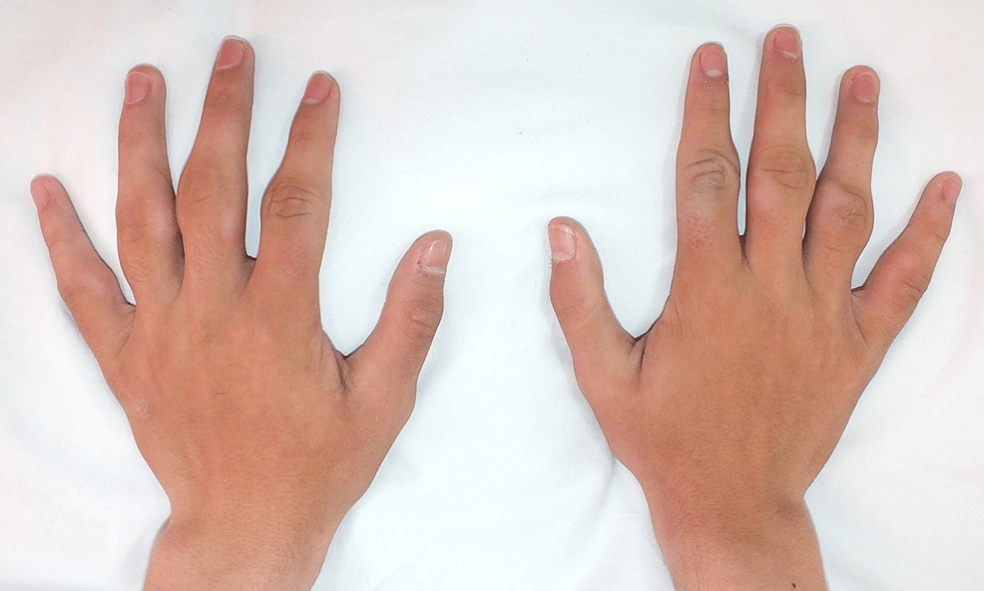
Physical examination Asymptomatic and chronic soft tissue enlargement of the fingers

A 16-year-old male, without any family history of rheumatologic illness, presented to our outpatient dermatology clinic with a two-and-a-half-year history of progressive and asymptomatic soft tissue enlargement of multiple fingers in both hands. He denied any rheumatological affliction, mechanical trauma, movement impairment, and consanguinity. He was previously treated with colchicine, nonsteroidal anti-inflammatory drugs, and prednisone, without improvement. On examination, we found a soft, fusiform, and subcutaneous enlargement of the third to fifth laterals and the second to fourth medial aspects of the proximal interphalangeal joints of both hands. No tenderness or loss of range of motion was seen (Figure 1).

**Figure 2:**
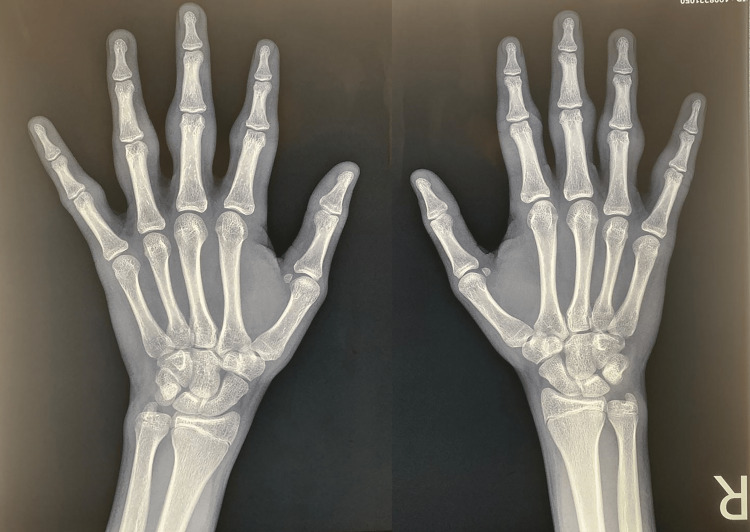
Radiograph Anteroposterior radiograph of both hands without bone and joint alteration

His complete blood count and basic metabolic panel results were normal, as well as his rheumatoid factor (RF), anti-cyclic citrullinated peptide (anti-CCP) antibodies, anti-nuclear antibodies (ANA), erythrocyte sedimentation rate, and C-reactive protein. A plain radiograph of both hands evidenced soft tissue swelling without bone involvement or loss of joint spaces (Figure 2).

**Figure 3:**
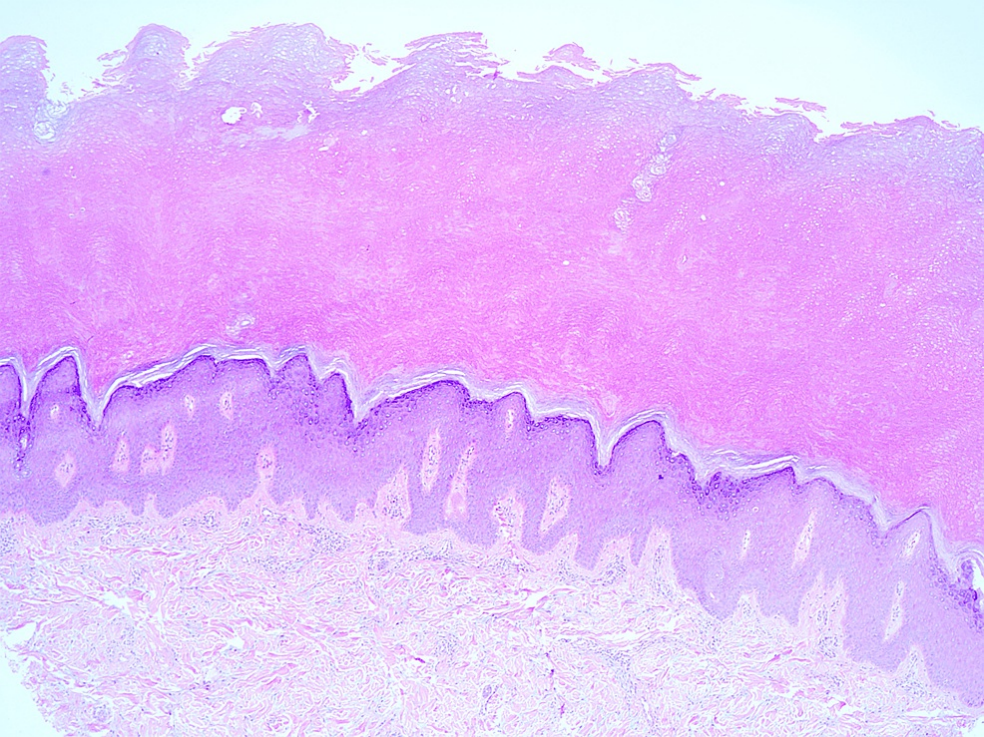
Histopathology image Orthokeratotic hyperkeratosis and thickened collagen bundles in the dermis. Stain hematoxylin and eosin magnification 10x

The histopathological study revealed orthokeratosis, acanthosis, and thickened collagen bundles in the dermis with a mild inflammatory infiltrate mainly composed of lymphocytes and deposition of mucin between collagen fibers (Figure 3). 

The patient history, clinical presentation of asymptomatic swelling of all the hand fingers, an unremarkable X-ray, and the histopathological findings of hyperkeratosis led to the diagnosis of PDD. Accordingly, conservative treatment with intralesional steroids was started for two months as the first-line treatment due to the following reasons: 1) The patient did not present any symptom associated to the swelling fingers, and 2) there was no limitation in daily activities.

## Discussion

PDD is a rare and benign digital fibromatosis characterized by asymptomatic, symmetrical, progressive, and fusiform swelling of the fingers [1,2]. It usually affects the medial and lateral sides of the proximal interphalangeal joints [4,5]. PDD is thought to be the consequence of excessive and repetitive mechanical trauma or due to hormonal dysfunction. The mechanical damage upregulates integrin β1, cytoskeleton p130Cas, transforming growth factor-beta 1 (TGF β1), and type I collagen. This repetitive trauma has been associated with neuropsychiatric disorders and athletic and occupational activities [1,2] . Regarding its hormonal dysfunction, it has been noted on in vitro models that androgens might stimulate fibroblasts and promote collagen synthesis and its deposition on different sites [1,2,6]. Its prevalence is unknown. Nevertheless, there is a recorded male-to-female ratio of 4:1, with a median age of 16 years at diagnosis [2]. The proximal interphalangeal joints are the most affected, but it might rarely affect the distal and metacarpophalangeal joints of the second to fourth digits in a bilateral fashion [1,2]. Rare cases of unilateral involvement have also been reported [7,8]. The thickening of the periarticular tissues mainly affects the lateral aspects and the dorsum of the joints, giving the fingers a fusiform appearance [8]. PPD is generally asymptomatic, but pain or tenderness have been reported in some cases [1].

Bardazzi et al. [9] classified all cases of PDD into five different types: 1) classic, where several fingers are affected, mostly in young males and related to microtraumas; 2) monopachidermodactily, where only one joint is involved; 3) transgrediens, where the enlargement extends toward the metacarpophalangeal joints and the dorsum of the hand; 4) familiar with a positive family history; and 5) associated with tuberous sclerosis or Ehlers-Danlos syndrome. In our case, the patient did not present any family member affected, and the physical examination was unremarkable besides the swelling of the fingers. A serological test to check the complete blood count, erythrocyte sedimentation rate, C-reactive protein, RF, anti-CCP antibodies, and ANA was performed, and the results were normal. 

Imaging studies showed soft tissue swelling or rarely bone atrophy or ventral and lateral subluxation of the third and fourth proximal interphalangeal joints [1,4]. Skin biopsy was performed to exclude other diagnosis. Although the findings are unspecific, orthokeratosis, acanthosis, increased number of fibroblasts, increased collagen deposits with an abnormal distribution of collagen types III and V, and mucin deposit were observed [1,7] .

The diagnosis of PDD is usually delayed from one to five years [5]. However, PDD is distinguished from juvenile inflammatory arthropathies and other related rheumatic diseases owing to the lack of osteoarticular involvement; functional impairment or skin changes, such as erythema, edema, or warmth; a painless course; and normal blood test. With other skin callosities of the fingers, such as the knuckle pads and/or pseudo-knuckle pads, they tend to affect the extensor and dorsal surfaces of the fingers rather than the radial and ulnar aspects in PDD, and they tend to be focal [4,7,8]. Because many PDD cases are wrongly diagnosed as juvenile idiopathic arthritis or rheumatoid arthritis, affected patients undergo unnecessary treatments. Other differential diagnoses include Thiemann disease, true knuckle pads, juvenile fibromatosis, Touraine-Solente-Golé syndrome, and hypertrophic pulmonary osteoarthropathy [6] . 

There is no accepted treatment for PDD. Intralesional glucocorticoids, surgery, and analogs of a tryptophan metabolite (tranilast) have been used with relative improvement [1,10]. A spontaneous remission can present after stopping physical stress [11]. In the present case, our patient was treated with intralesional triamcinolone with mild improvement.

## Conclusions

PDD is a rare fibromatosis characterized by fusiform swelling fingers of presumed traumatic and hormonal etiology. It is of outmost importance to report its pediatric cases to record epidemiological data and to establish diagnostic and prognostic factors that could shorten its delayed diagnosis. Here, we presented a case of PDD with improvement of the swelling and movements of the affected fingers. However, there are still no definitive treatment guidelines.
